# Sensitivity and correlation of hypervariable regions in 16S rRNA genes in phylogenetic analysis

**DOI:** 10.1186/s12859-016-0992-y

**Published:** 2016-03-22

**Authors:** Bo Yang, Yong Wang, Pei-Yuan Qian

**Affiliations:** Division of Life Sciences, Hong Kong University of Science and Technology, Clear Water Bay, Hong Kong; Sanya Institute of Deep Sea Science and Engineering, Chinese Academy of Sciences, San Ya, Hai Nan China

**Keywords:** 16S rRNA, 16S rRNA gene, Variable regions, Phylogenetic, Geodesic distance, Primer

## Abstract

**Background:**

Prokaryotic 16S ribosomal RNA (rRNA) sequences are widely used in environmental microbiology and molecular evolution as reliable markers for the taxonomic classification and phylogenetic analysis of microbes. Restricted by current sequencing techniques, the massive sequencing of 16S rRNA gene amplicons encompassing the full length of genes is not yet feasible. Thus, the selection of the most efficient hypervariable regions for phylogenetic analysis and taxonomic classification is still debated. In the present study, several bioinformatics tools were integrated to build an *in silico* pipeline to evaluate the phylogenetic sensitivity of the hypervariable regions compared with the corresponding full-length sequences.

**Results:**

The correlation of seven sub-regions was inferred from the geodesic distance, a parameter that is applied to quantitatively compare the topology of different phylogenetic trees constructed using the sequences from different sub-regions. The relationship between different sub-regions based on the geodesic distance indicated that V4-V6 were the most reliable regions for representing the full-length 16S rRNA sequences in the phylogenetic analysis of most bacterial phyla, while V2 and V8 were the least reliable regions.

**Conclusions:**

Our results suggest that V4-V6 might be optimal sub-regions for the design of universal primers with superior phylogenetic resolution for bacterial phyla. A potential relationship between function and the evolution of 16S rRNA is also discussed.

**Electronic supplementary material:**

The online version of this article (doi:10.1186/s12859-016-0992-y) contains supplementary material, which is available to authorized users.

## Background

As the major players in almost all environments explored, bacteria contribute immensely to global energy conversion and the recycling of matter. Thus, profiling of the microbial community is one of the most important tasks for microbiologists to explore various ecosystems. However, our understanding of the kingdom Bacteria remains limited because most bacteria cannot be cultured or isolated under laboratory conditions [1]. In the past few decades, DGGE (Denaturing gradient gel electrophoresis) [2], T-RFLP (Terminal restriction fragment length polymorphism) [3], FISH (fluorescent in situ hybridization) [4] and Genechips [5] were used as mainstream methods in studies of bacterial communities and diversity until the development of high-throughput sequencing technology. Recently, meta-genomic methods provided by next-generation sequencing technology such as Roche 454 [6, 7] and Illumina [8] have facilitated a remarkable expansion of our knowledge regarding uncultured bacteria [7].

The 16S rRNA gene sequence was first used in 1985 for phylogenetic analysis [9]. Because it contains both highly conserved regions for primer design and hypervariable regions to identify phylogenetic characteristics of microorganisms, the 16S rRNA gene sequence became the most widely used marker gene for profiling bacterial communities [10]. Full-length 16S rRNA gene sequences consist of nine hypervariable regions that are separated by nine highly conserved regions [11, 12]. Limited by sequencing technology, the 16S rRNA gene sequences used in most studies are partial sequences. Therefore, the selection of proper primers is critical to study bacterial phylogeny in various environments.

An early study has shown that the use of different primers might result in different DGGE patterns [13]. Recent studies utilizing high throughput technology have also demonstrated that the use of suboptimal primer pairs results in the uneven amplification of certain species, causing either an under- or over-estimation of some species in a microbial community [10–12, 14]. Although several studies have focused on optimal primer pairs or, equivalently, optimal variable regions for the study of bacterial communities [15–17], they utilized synthetic microbial communities and the taxa that were chosen to conduct those experiments would largely influence the final results. Consequently, the use of different sequencing technologies and targeting of different sub-regions of 16S rRNA genes will result in a distinct composition of a given microbial community. However, till now there was few study focusing on comparing the phylogenetic sensitivity of the 16S rRNA sub-regions.

Phylogenetic trees are widely used to elucidate systematic relationships between different species, in particular the novel microbial lineages [9, 18–20]. However, strategies to determine relationships between different 16S rRNA sub-regions in terms of phylogenetic resolution remain questionable. The correlation of the different hypervariable regions may be inferred from the geodesic distance of phylogenetic trees that are constructed based on the sequences of different regions. The topological similarity between phylogenetic trees may be estimated by a geodesic algorithm that can project the node structure of a tree into a multi-dimensional model [21]. The geodesic distance has been used to quantify discrepancies between trees [22, 23]. A recent study took advantage of this method to quantitatively compare phylogenetic trees that were reconstructed using different essential genes [1]. Other than 16S r RNA genes, concatenated essential marker genes are preferred for phylogenetic analyses [24]. However, as suggested by the pairwise geodesic distance, the topology of the tree based on the amino acids of translation initiation factor 2 (IF2) is highly similar to that obtained with the concatenated marker sequences [1], suggesting that IF2 can be applied alone for phylogenetic reconstruction and roughly reflects genetic relationships using all of the other essential genes. Using the geodesic algorithm, it is possible to quantify the sensitivity of 16S rRNA variable regions compared with the full-length 16S rRNA sequences. These quantitative data also permit the exploration of correlative relationships between different sub-regions of 16S rRNA in terms of the phylogenetic resolution. In the present study, we designed an *in silico* pipeline to evaluate the phylogenetic resolution of different variable regions in 16S rRNA genes.

## Methods

### Data source and pre-treatment

**Fig. 1:**
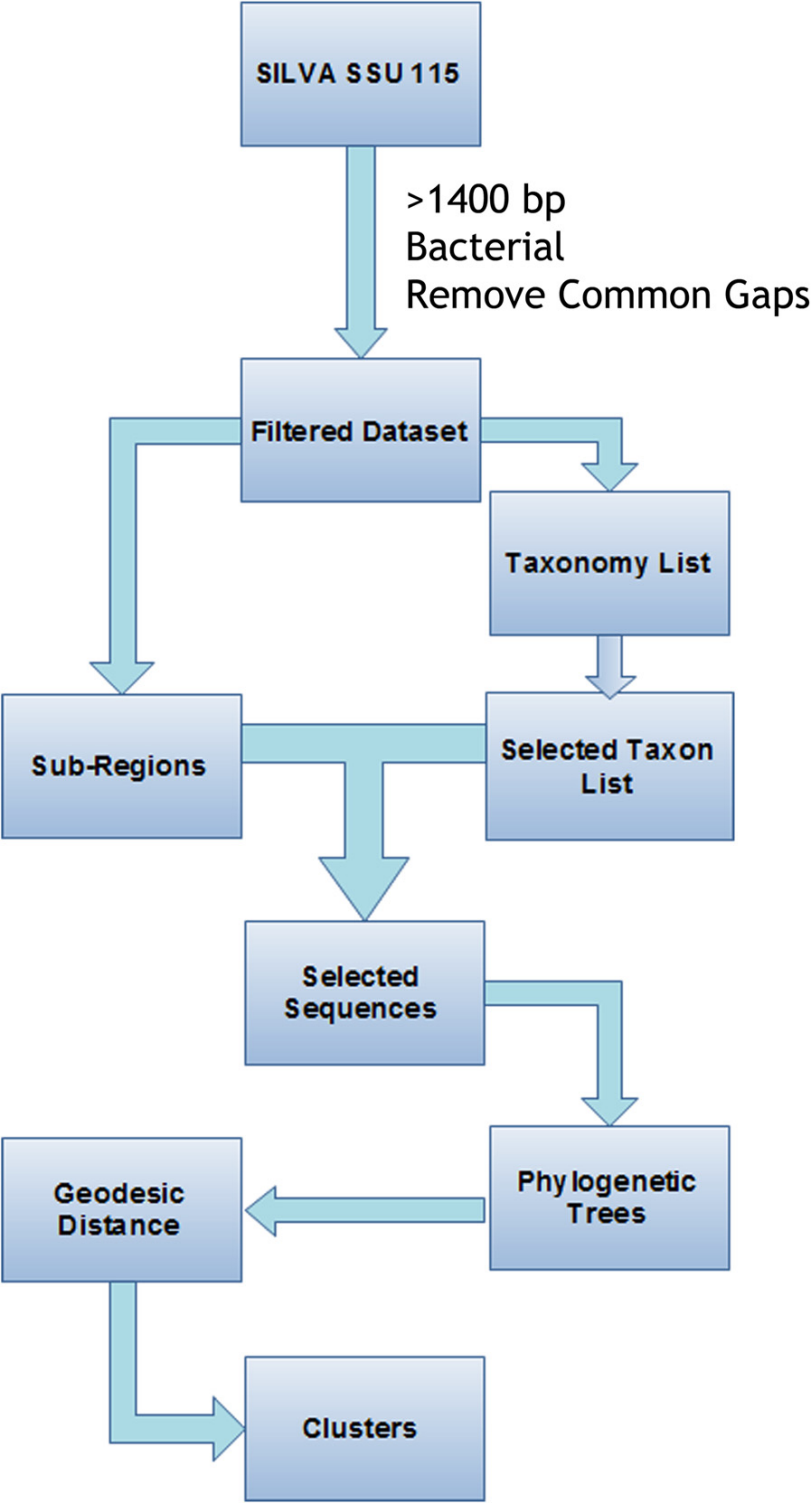
Workflow of the data processing. As described in Materials and methods, the sequences downloaded from the SILVA database were filtered, randomly selected and grouped. Phylogenetic trees were then built, and geodesic distances were calculated

The pre-aligned and truncated SILVA Ref 115 16S rRNA NR99 dataset was downloaded from SILVA online database as a primary dataset [25]. The original downloaded dataset from SILVA contains 479,726 nearly full-length 16S/18S sequences of Archaea, Bacteria and Eukaryota. The pre-processed dataset contained 79,096 sequences from the kingdom Bacteria. The following filtration criteria were applied to the primary dataset: 1) longer than 1400 bp; and 2) SILVA annotated taxa. The full-length bacterial 16S rRNA dataset was then processed as described in Fig. 1. SILVA database sorted organelles sequences into Bacterial kingdom, so the organelles sequences were manually processed.

### Definition of sub-regions

Referring to the previous studies [11, 12], 10 markers located in conserved regions of the 16S rRNA genes were selected to divide the full-length aligned 16S rRNA sequences into nine sub-regions (Additional file 1: Table S1). Each region starts with a conserved sequence, and the remainder is a downstream variable sequence. The tenth conserved region was the termination marker and was removed from the ninth sub-region. The breakpoints of each region in the aligned data are shown in Table 1. The sub-regions were sequentially marked from V1 to V9. The filtered dataset was divided into 10 files: nine for the V1-V9 sub-regions, and one for the combination of all of the sub-regions (VT). After division of the sub-regions, most of the V1 and V9 sub-regions were found to be incomplete. As a result, V1 and V9 were not included in the subsequent analysis.

**Table 1:** Positions of the hypervariable sub-regions of the 16S rRNA sequences

Region	Start position	End position	Start postion (E. Coli)	End position (E. Coli)
V1	8	789	8	96
V2	790	2697	97	306
V3	2698	4069	307	487
V4	4070	7044	488	746
V5	7045	9533	747	885
V6	9534	10454	886	1029
V7	10455	12258	1030	1180
V8	12259	13597	1181	1372
V9	13598	14371	1373	1468
VT	8	14371	8	1468

### Selection of representative sequences

Sequences in the filtered dataset were annotated with taxonomic ranks by SILVA [25]. A taxonomic list with SILVA accession IDs was first extracted from the filtered dataset. The following criteria were then applied to select representative taxa from the taxonomic list: 1) three sequences in each phylum were randomly selected, but phyla with less than three sequences were discarded; 2) three sequences from different sub-levels within a phylum were preferred; 3) sequences belonging to chloroplasts and mitochondria were avoided; 4) three sequences were selected from different classes under five subphyla of the Proteobacteria. The phylum Proteobacteria has a huge number of sequences in the filtered dataset, and therefore, the five major groups (alpha, beta, gamma, epsilon and delta) of Proteobacteria were treated as subphyla. For example, if a proteobacterial subphylum contained five classes, the first step consisted of the random selection of three classes in this subphylum followed by the random collection of full-length sequences from each of the selected classes. By abiding to these rules, 89 taxonomic lists were produced. Each of the lists corresponded to a sequence file containing 93 sequences from 31 phyla and 15 sequences from Proteobacteria, providing a total of 108 sequences. For each of the sequence files, individual sub-regions V2-V8 of the 16S sequences were extracted to create new sequence files. Finally, a total of 76,896 sequences were distributed in a three-dimensional array consisting of 89 taxonomic lists, 108 full-length 16S sequences and eight regions (V2-V8 & VT). All the data could be accessed in SILVA database with the Sequence IDs provided in Additional file 2.

### Construction of phylogenetic trees

The phylogenetic relationships of the 16S sub-regions were inferred using the Bayesian algorithm. The Bayesian MCMC analysis program BEAST (version 1.8.0) [26] was utilized to build phylogenetic trees. For a given taxonomic list, the aligned 16S rRNA sequence files in FASTA format from the seven sub-regions were first converted into Nexus files. Using the BEAuti software in the BEAST package, the nexus files were annotated with the GTR substitution model and the Gamma & Invariant sites heterogeneity model. Next, these files were processed using BEAST software to build phylogenetic trees. The trees then were annotated using the TreeAnnotator software in the BEAST package with parameter “-burnin 2000”, which removed the first 20 % of the trees constructed by BEAST and provided a more stable result. The annotated trees were then converted to Newick format for geodesic analysis. For each taxonomic list, seven trees were built for the different sub-regions. A Bayesian tree was also constructed for the VT for the following pairwise comparison. In this step, a total of 712 trees were built (89 groups, each group generated eight trees, each tree contained 108 sequences/taxa).

### Geodesic distance and clustering

The geodesic distance between Bayesian phylogenetic trees was calculated by software based on the GTP algorithm [22, 23]. The topological similarity of the trees using the sub-regions and VT could be estimated by their relationship in agglomerative hierarchical clustering (AHC). There were 28 pairwise geodesic distances between the sub-regions (including VT) in a taxonomic list. The distance matrix was then applied for AHC clustering analysis using the XLSTAT software. To calculate the frequencies for the nodes of the clustering structure, AHC analysis was performed for the other taxonomic lists. The clustering results of the 89 lists were converted manually into trees in Newick format. Using the Consensus program in the PHYLIP (version 3.6) package [27], an ultimate clustering relationship with supportive probabilities for the nodes was generated. In this step, totally 2492 geodesic distances were calculated (89 groups, each group had *C*_8_^2^ = 28 geodesic distances).

## Results and discussion

### Geodesic distance between VT and sub-regions

**Fig. 2:**
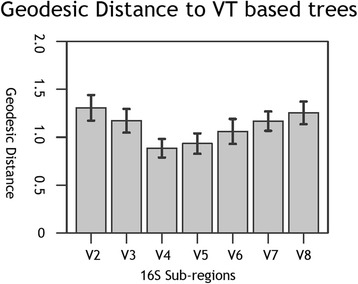
Geodesic distance between trees based on sub-regions and trees based on VT

The geodesic distance between sub-regions and VT is shown in Fig. 2. Because 89 taxonomic groups were used for the analysis, the average and standard deviations for the distance values are also displayed. The results demonstrated that the pairwise distance of V4-VT was the smallest distance, which indicated that the topology of the trees using V4 most closely resembled that using VT. V5 and V6 were adjacent to V4 in terms of the geodesic distance to VT. The geodesic distances between trees based on merged sub-regions V2-3-4, V3-4-5, V4-5-6, V5-6-7 V6-7-8 and RT trees were also calculated (Additional file 3: Figure S1). The results also supported that V4-5-6 was the optimal region combination. In contrast, the pairwise distances of V2-VT and V8-VT were larger than the others, indicating that the phylogenetic relationships inferred from the V2 and V8 sub-regions were very different from the VT-based results.

By calculating the geodesic distance between different regions, the phylogenetic relationships based on the V4 sequences were closest to those based on the full-length sequences. This result suggests that V4 ranks first in sensitivity as a marker for bacterial and phylogenetic analysis, which is consistent with previous taxonomic results obtained using the RDP (Ribosomal Database Project) classifier [28]. However, the RDP classifier method, which has been repeated using the dataset in the present study, fails to demonstrate the best performance of V4 at phylum level. The sequences used in this project were also analyzed by the RDP classifier in QIIME pipeline, the results showed no significant difference in order level (Additional file 4: Figure S2). Therefore, using geodesic distance to compare the performance of different regions would be more sensitive. In addition, V1-V3 were also highly recommended by some previous studies [15], but our results demonstrated poor performance for V2 and V3 in terms of the phylogenetic analysis.

### Geodesic distance-based AHC of sub-regions

**Fig. 3:**
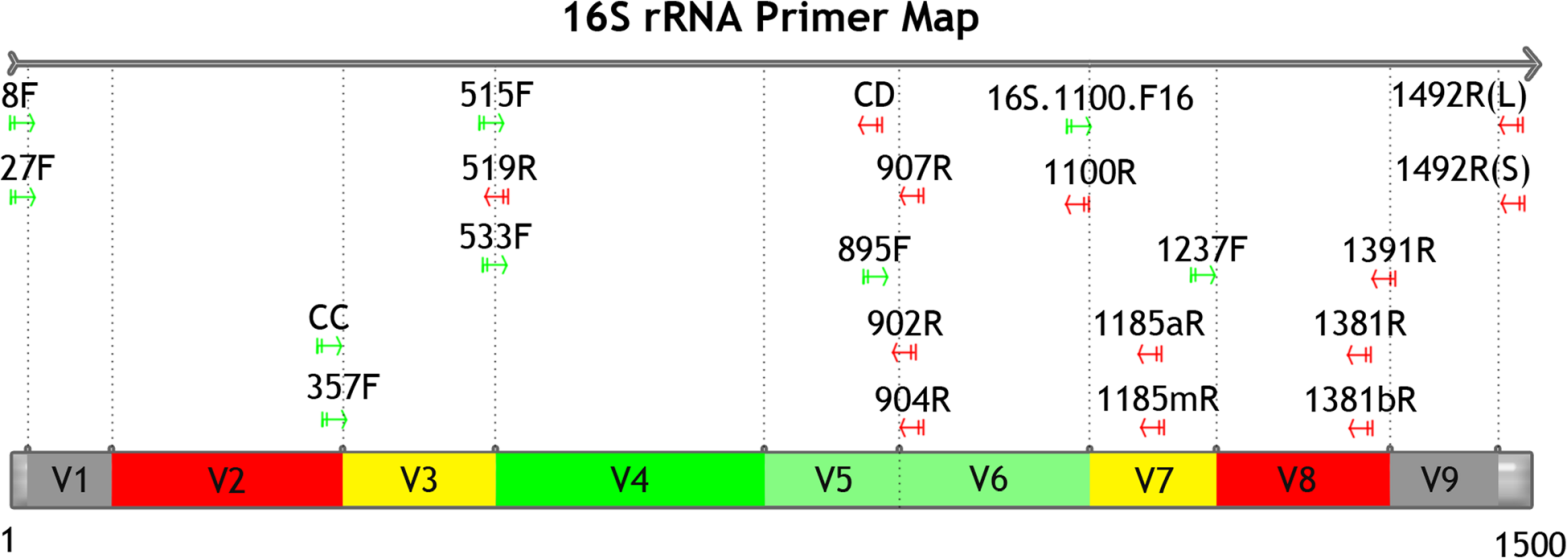
Illustration of different variable regions. Red regions (V2, V8) have a poor phylogenetic resolution at the phylum level. Green regions (V4, V5, V6) are associated with the shortest geodesic distance, which suggests that they may be the best choice for phylogeny-related analyses and the phylogenetic analysis of novel bacterial phyla. The figure refers to the primer map from Lutzonilab (http://lutzonilab.org/16s-ribosomal-dna/). Use of this information was approved by the original authors of the website

**Fig. 4:**
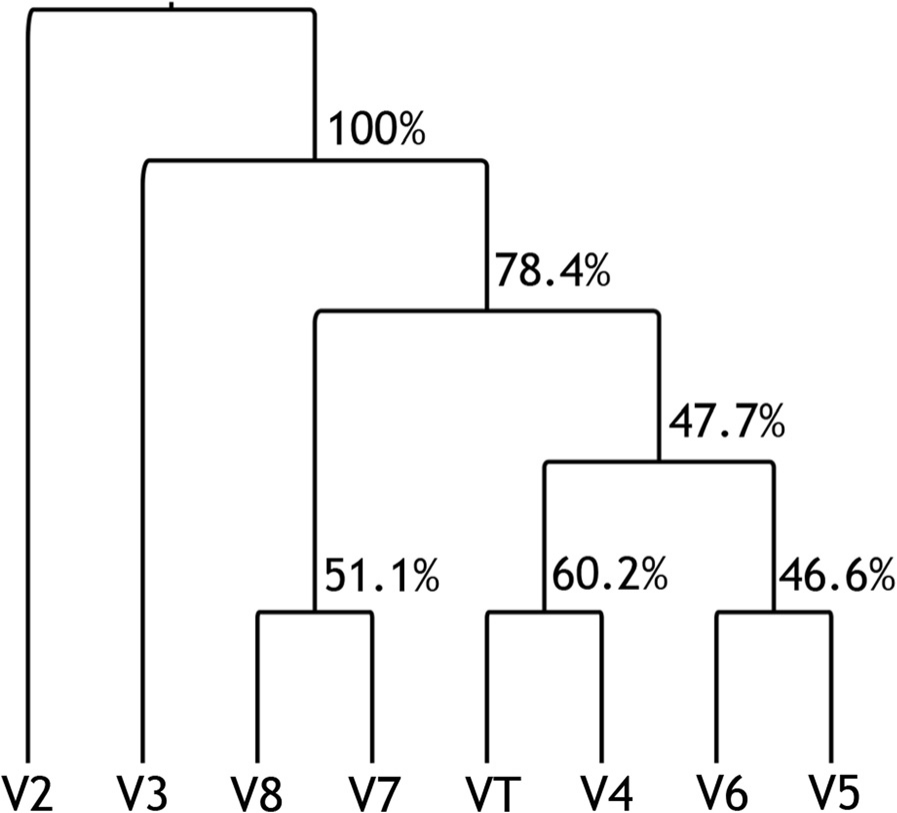
AHC results for different regions based on the geodesic distances of the phylogenetic trees

Using the geodesic distance matrix, AHC analysis was performed to reflect the correlative relationships between the sub-regions in terms of the phylogenetic resolution. The consensus AHC cluster showed that V2 and V3 were always the outgroups in the AHC, which was supported by high probabilities (> 70 %). The other nodes of the clusters, such as V8-V7, V6-V5, and (VT-V4)-(V6-V5), were not highly supported. However, evidence for the relationships between different regions was still obtained, thus serving as an indicator of the correlations between different sub-regions. The closest relationship between V4 and VT was again illustrated by the AHC coupled with a probability of 60.2 %. Therefore, V4 was the best sub-region for the phylogenetic study, particularly at the phylum level. After combining the geodesic distance results and AHC pattern, we sorted the regions into three groups in terms of the phylogenetic resolution (Fig. 3) [9, 20, 29–32]. Class I, which included V4, V5 and V6 (Fig. 4), had the highest sensitivity and has been suggested to represent the optimal sub-regions for phylogenetic studies. V3 and V7 are within Class II (yellow in Fig. 3) and showed moderate sensitivity. Class III, which was represented by V2 and V8, was not used for phylogenetic resolution at the phylum level or for phylogenetic analyses of diverse communities although Class III may still be suitable for the phylogenetic analysis and possibly for classification of microbes from the same classes or families. The regions ranging from 515 F to 1100R or from V4 to V6 were more suitable for studies of extreme environments with novel bacterial lineages.

**Fig. 5:**
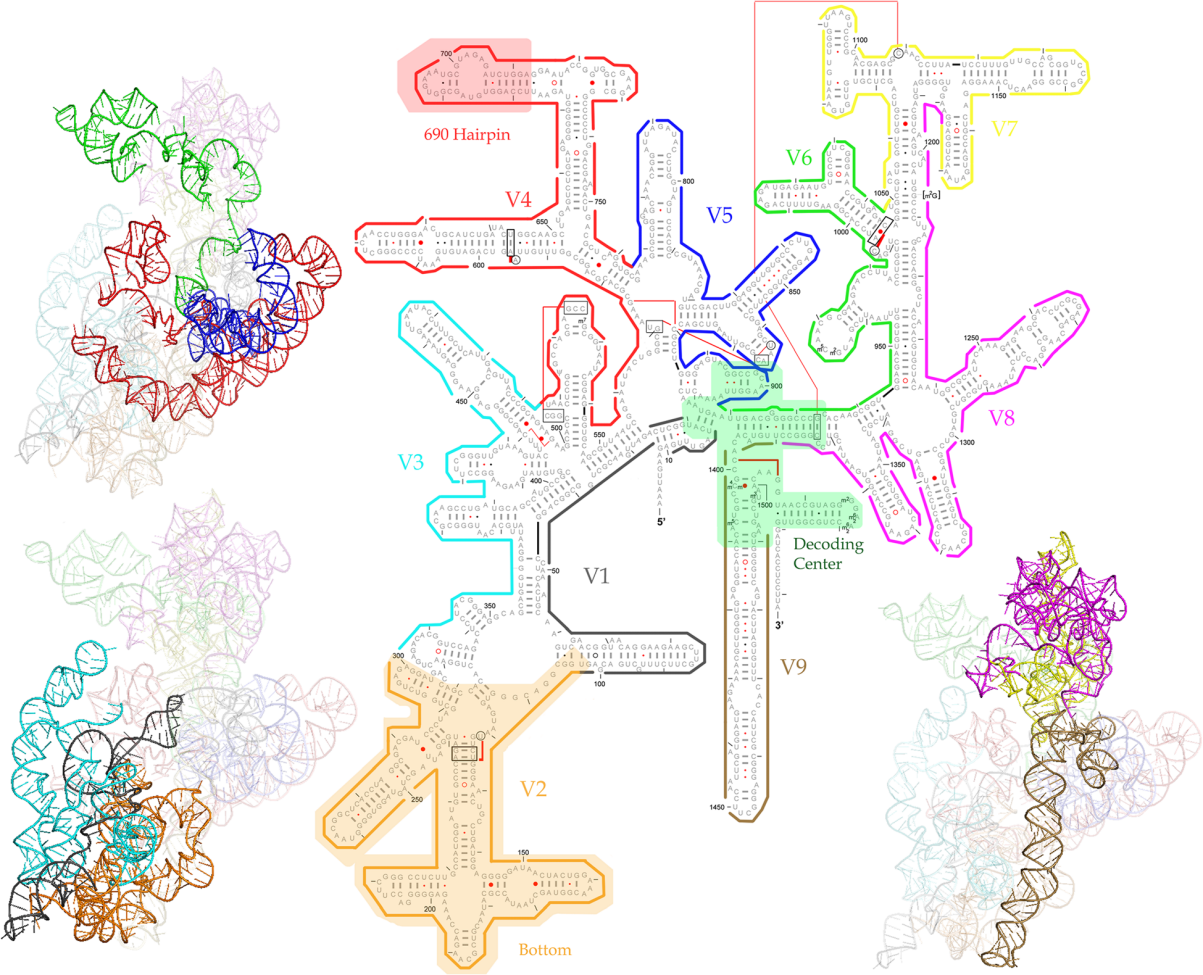
The 2D-3D structures of the 16S rRNA gene. Individual regions are identified by the same color in both the 2D and 3D structures. Some important structures are colored with blocks

The underlying cause of the correlation between different sub-regions in terms of phylogenetic resolution remains unknown. Because 16S rRNA itself carries out the process of gene translation, it is quite interesting to potentially connect these regions with the 3D structure and functioning sites (Fig. 5). Class I regions spanning V4, V5 and V6 are the major functional parts of the 16S rRNA because they encompass the ‘690 hairpin’ [33–35] and decoding center [36, 37]. The 690 hairpin is a highly conserved loop in all three phylogenetic domains located at the V4 region of 16S rRNA [34, 38]. This region has been reported to be related to P-site-bound tRNA, S11 binding, IF3 binding and RNA-RNA interactions with the 790 loop of the 16S rRNA and domain IV of the 23S rRNA [24, 35, 39–46]. The decoding center is also involved in V9, but it was not considered in the present study. Therefore, whether the positions in the decoding center determine the phylogenetic resolution could not be confirmed herein. The Class II regions V3 and V7 are peripheral to the two functional centers of the 16S rRNA [36, 37]. Important functional roles have not yet been confirmed. Class (III) regions V2 and V8 are located at the bottom and top, respectively, of the 3D structure of 16S rRNA [36, 37]. They may serve as structural stabilizers of the 16S rRNA, but no functional importance has been reported to date. This observation is similar to the debate over the association between the evolutionary rate and gene dispensability [47–49]. According to this theory, genes with a high dispensability may have evolved slowly. In contrast, the differences in less important regions, such as Class II, may occur at lower taxonomic levels. Similarly, in our study, the functions associated with Class I regions might evolve at a lower rate and be more stable than the other variable regions. As a result, these regions could allow the realization of a more stable phylogenetic topology among the diverse bacterial phyla. Class II and Class III regions are less conserved and display more polymorphisms that may occur only at lower taxonomic levels. Thus, these sub-regions are less sensitive as markers for the phylogenetic resolution of a novel lineage within a community at the phylum level. However, the functioning sites are usually quite short in comparison with the whole sub-region and thus, it is questionable whether the several conserved sites determine the topology of a phylogenetic tree consisting of 32 different phyla.

## Conclusions

In the present study, we evaluated the sensitivity of different 16S rRNA sub-regions as biomarkers of different bacterial phyla using the geodesic distance method and the consensus AHC method. A combination of V4-V6 was determined to represent the optimal sub-regions for the bacterial phylogenetic study of new phyla. Furthermore, for the first time, we briefly evaluated the correlation of different sub-regions of 16S rRNA in terms of the phylogenetic resolution, which might suggest a relationship between the function and evolution of 16S rRNA genes.

### Ethics

There were no human, human data or animals involved in this study.

## Additional files

Additional file 1: Table S1. Conserved Markers used to identify sub-regions. (DOCX 12 kb) Additional file 2: This compressed file contains all the SILVA *SeqIDs* and taxonomy of the data we processed in this study. All the sequences could be accessed from SILVA with the *SeqIDs*. (ZIP 358 kb) Additional file 3: Figure S1. Geodesic distance between merged sub-regions tree and RT trees. (TIF 1981 kb) Additional file 4: Figure S2. QIIME analysis of dataset in the manuscript. For each sub-region, we merged 89 datasets, each contained 108 sequences, into one multi-sequence fasta file. Then different barcodes were manually for different sub-regions. All the following analysis were following the standard QIIME pipeline with default parameters. The results showed no significant difference between different regions. (TIF 249 kb)
