# Supplementary material for: Sensitivity and correlation of hypervariable regions in 16S rRNA genes in phylogenetic analysis
Source: BMC Bioinformatics. 2016 Mar 22;17:135. doi: 10.1186/s12859-016-0992-y (PMC4802574; doi:10.1186/s12859-016-0992-y)
Supplement: Additional file 1: Table S1. — Conserved Markers used to identify sub-regions. (DOCX 12 kb) [file 12859_2016_992_MOESM1_ESM.docx]

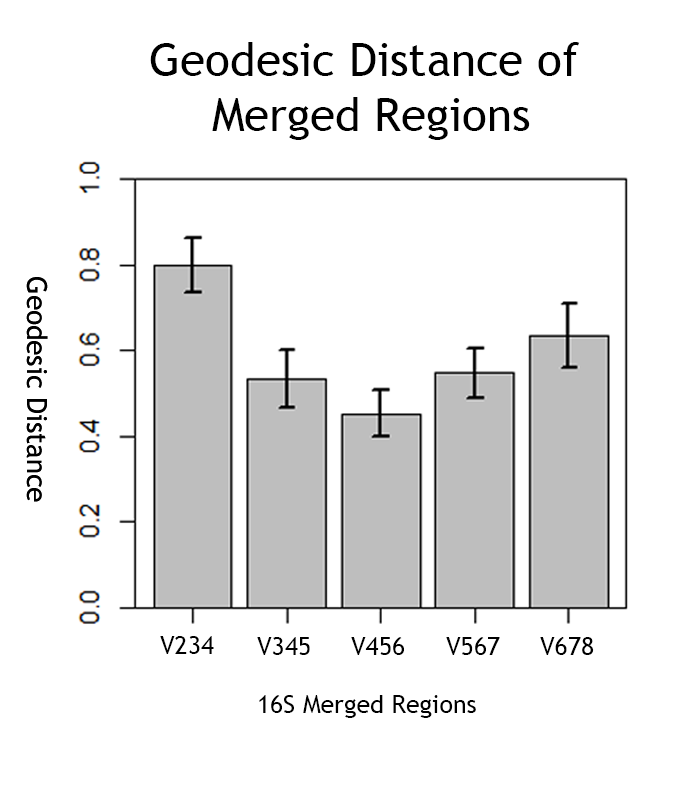


**Figure S1.** Geodesic distance between merged sub-regions tree and RT trees.


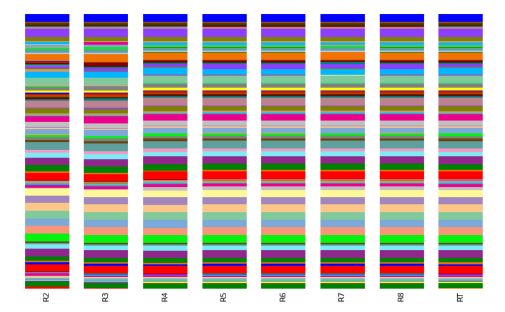


**Figure S2.** QIIME analysis of dataset in the manuscript. For each sub-region, we merged 89 datasets, each contained 108 sequences, into one multi-sequence fasta file. Then different barcodes were manually for different sub-regions. All the following analysis were following the standard QIIME pipeline with default parameters. The results showed no significant difference between different regions.

**Table S1.** Conserved Markers used to identify sub-regions.

| Conserved Marker | Sequence |
| --- | --- |
| 1 | AGAGTTTGATCATGGCTCA |
| 2 | GGCGVACGGGTGAGTAA |
| 3 | CAYTGGRACTGAGACACGGYCC |
| 4 | GGCTAACTHCGTGCCAGCAGC |
| 5 | CGAAAGYGTGGGKAKCRCAGG |
| 6 | ACTCAAAKGAATTGACGGGGRC |
| 7 | GTGSTGCATGGYTGTCGTCAGCT |
| 8 | GGAAGGYGGGGAYGACGTCAA |
| 9 | TGTACACACCGCCCGTCACACAC |
| End of 16S | AAGTCGTAACAAGGTAACCGTA |

File:

**SeqIDs.zip**

This compressed file contains all the SILVA SeqIDs and taxonomy of the data we processed in this study. All the sequences could be accessed from SILVA with the SeqIDs.
