# Supplementary figures and images for: Sensitivity and correlation of hypervariable regions in 16S rRNA genes in phylogenetic analysis
Source: BMC Bioinformatics. 2016 Mar 22;17:135. doi: 10.1186/s12859-016-0992-y (PMC4802574; doi:10.1186/s12859-016-0992-y)

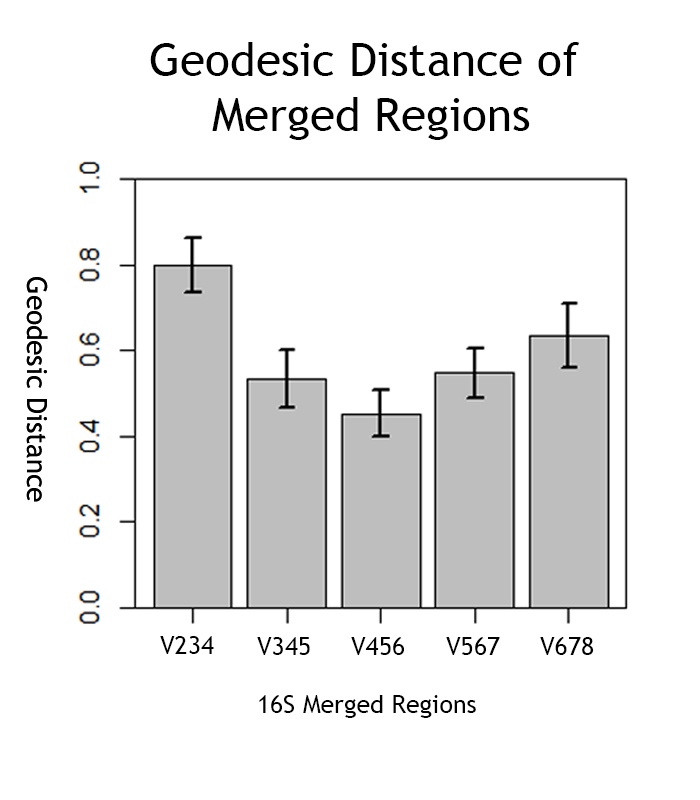

Supplement: Additional file 3: Figure S1. — Geodesic distance between merged sub-regions tree and RT trees. (TIF 1981 kb) [file 12859_2016_992_MOESM3_ESM.tif]

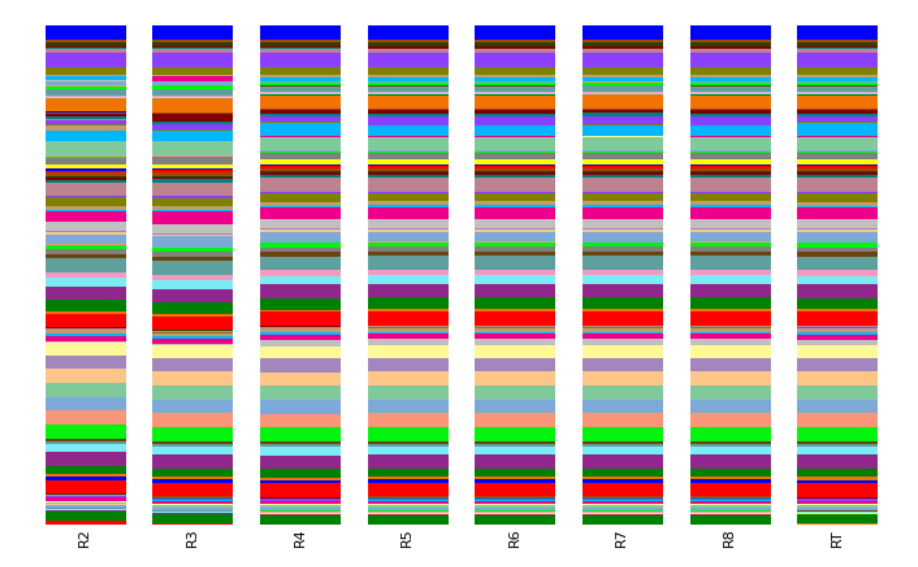

Supplement: Additional file 4: Figure S2. — QIIME analysis of dataset in the manuscript. For each sub-region, we merged 89 datasets, each contained 108 sequences, into one multi-sequence fasta file. Then different barcodes were manually for different sub-regions. All the following analysis were following the standard QIIME pipeline with default parameters. The results showed no significant difference between different regions. (TIF 249 kb) [file 12859_2016_992_MOESM4_ESM.tif]
